# Percutaneous dilatational tracheostomy with single use bronchoscopes versus reusable bronchoscopes – a prospective randomized trial (TraSUB)

**DOI:** 10.1186/s12871-022-01618-4

**Published:** 2022-04-02

**Authors:** Pischtaz Adel Tariparast, Andrés Brockmann, Rainer Hartwig, Stefan Kluge, Jörn Grensemann

**Affiliations:** Department of Intensive Care Medicine, University Medical Center Hamburg-Eppendorf, Martinistraße 52, 20246 Hamburg, Germany

## Abstract

**Background:**

Apart from conventional reusable bronchoscopes, single-use bronchoscopes (SUB) were recently introduced. Data suggest that SUB might prevent from the risk of cross contamination (i.e. multiresistant pathogens, SARS CoV-2) and save costs. We aimed to investigate visualization, ventilation, handling characteristics, changes in patients’ gas exchange, and costs associated with both types of bronchoscopes during percutaneous dilatational tracheostomy (PDT).

**Methods:**

In this prospective, randomized, noninferiority study, 46 patients undergoing PDT were randomized 1:1 to PDT with SUB (Ambu aScope) or reusable bronchoscopes (CONV, Olympus BF-P60). Visualization of tracheal structures rated on 4-point Likert scales was the primary end-point. Furthermore, quality of ventilation, device handling characteristics, changes in the patients’ gas exchange, pH values, and costs were assessed.

**Results:**

Noninferiority for visualization (the primary endpoint) was demonstrated for the SUB group. Mean visualization scores (lower values better) were 4.1 (95% confidence intervals: 3.9;4.3) for SUB vs. 4.1 (4.0;4.2) for CONV. Noninferiority of ventilation (estimated by minute volume and SpO_2_) during the procedure could be shown as well. Mean score was 2.6 (2.0;3.1) for SUB vs. 2.4 (2.1;2.7) for CONV (lower values better). No significant differences regarding handling (SUB: 1.2 (1.0;1.4), CONV: 1.3 (1.1;1.6)), blood gas analyses and respiratory variables were found. Cost analysis in our institution revealed 93 € per conventional bronchoscopy versus 232.50 € with SUB, not considering an estimate for possible infection due to cross-contamination with the reusable device.

**Conclusion:**

In our study, visualization and overall performance of the SUB during PDT were noninferior to reusable bronchoscopes. Therefore, PDT with SUB is feasible and should be considered if favored by individual institution’s cost analysis.

**Trial registration.:**

ClinicalTrials.gov, NCT03952247. Submitted for registration on 28/04/2019 and first posted on 16/05/2019.

## Background

In 1966, the first flexible bronchoscope was introduced [1]. Since then, bronchoscopy has evolved into a routine procedure in intensive care units, readily available at the bedside. Until today, bronchoscopies are usually performed with reusable bronchoscopes that undergo reprocessing after use, but the cost-effectiveness has been questioned due to costs of maintenance, decontamination, and disinfection [2]. Furthermore, reusable devices carry the risk of infections due to cross-contamination [3]. Single-use bronchoscopes with a suctioning channel are now available for routine bronchoscopies at intensive care units and may be considered advantageous as maintenance, repair, and decontamination are not required [4]. Furthermore, during the current COVID-19 pandemic, single-use bronchoscopes are regarded as very useful to further reduce transmission risks [5].

Percutaneous dilatational tracheostomy (PDT) is a standardized procedure often performed in critically ill patients requiring long-term ventilation to facilitate weaning from ventilation [6] and has widely replaced surgical tracheostomy [7]. The PDT technique involves the puncture of the trachea using a modified Seldinger technique, i.e. dilating the tracheostomy tract by a guidewire. Simultaneous bronchoscopy facilitates the verification of the correct tracheotomy site and ensures the correct intratracheal placement of the guidewire, the dilator, and the tracheal cannula [8]. Consequently, bronchoscopically guided positioning of the PDT devices minimizes the risk of complications, particularly posterior tracheal wall injury [9]. Therefore, bronchoscopy guided PDT has been adopted by the majority of intensivists [7, 10].

Until today there are no data regarding the quality and handling characteristics of single-use bronchoscopes compared to their corresponding reusable counterparts during PDT. To recommend their use in PDT, a thorough evaluation versus reusable bronchoscopes is required. Therefore, we investigated single-use bronchoscopes (Ambu® aScope™ 4 Broncho, Ambu A/S, Ballerup, Denmark) in comparison to conventional reusable bronchoscopes for PDT. We assessed the visualization during PDT as well as ventilation and handling characteristics, changes in patients’ gas exchange and pH values in a prospective, randomized, noninferiority study. Furthermore, we assessed the costs associated with both types of bronchoscopes.

## Methods

### Study design

The TraSUB trial was a prospective, randomized study with a 1:1 allocation ratio conducted in the Department of Intensive Care Medicine at the University Medical Center Hamburg-Eppendorf, Germany consisting of twelve intensive care units treating adult patients with a total of 140 beds. The Ethics Committee of the Hamburg Chamber of Physicians, Germany, approved the study (reference PV5981; March 25, 2019). Written informed consent was obtained from the patients’ legal guardians. All methods were carried out in accordance with relevant guidelines and regulations. The study was registered with ClinicalTrials.gov, identifier: NCT03952247 (submitted for registration on 28/04/2019 and first posted 16/05/2019).

### Participants

All ventilated patients in the participating intensive care units were assessed for eligibility. Patients were eligible if they were at least 18 years old and required PDT for long-term ventilation and had a Cormack-Lehane score < 3. Screening and enrollment of patients was done by the authors (AB and PAT).

### Interventions

#### Tracheostomy

All patients underwent PDT with the Ciaglia single-step dilator technique (Ciaglia Blue Rhino^®^ G2; Cook Medical, Bloomington, IN, USA) in a standardized technique [11]. During the intervention, patients were mechanically ventilated in a pressure-controlled mode (bilevel positive airway pressure, Evita V500, Drägerwerk, Lübeck, Germany) via an orally placed endotracheal tube. Patients were anesthetized with propofol and/or midazolam and sufentanil. Rocuronium was used for muscle relaxation. A brief description of our tracheostomy protocol has been published before [12].

To reduce the risk of airway complications during and following tracheostomy, patients with a Cormack and Lehane score ≥ 3 were excluded and received surgical tracheostomy [13] according to our local protocol to ensure that in cases of an accidental extubation during tracheostomy, the airway could easily be reestablished under direct laryngoscopy. The trachea was mostly cannulated between the second and third tracheal cartilage after an optional blunt dissection of the subcutaneous tissue. All patients received an oxygenation with an inspiratory fraction of oxygen of 1 throughout the procedure. Tracheostomies were performed by senior physicians with a specialization in intensive care medicine.

#### Bronchoscopic procedure

In patients randomized to the SUB group, an Ambu^®^ aScope™ 4 Broncho (size: “regular”) with an outer diameter of 5.0 mm, a suctioning channel diameter of 2.2 mm, and a field of view of 85° was used. A conventional reusable bronchoscope was available as a backup safety measure and could be used at the treating physician’s discretion. In patients randomized to the CONV group, a reusable fiberoptic scope (Olympus BF-P60, Olympus Medical Systems Corp., Tokyo, Japan) with an outer diameter of 4.9 mm, a suctioning channel diameter of 2.2 mm, and a field of view of 120° was used. The fiberoptic scope was connected to a monitor (Olympus Medical Systems Corp.). During bronchoscopy, the endotracheal tube was retracted until the cricoid cartilage was visible. For assessment of the bronchoscope handling characteristics (rotation of the device, flexion of the tip of the bronchoscope, difficulty to advance and pull back the bronchoscope) the bronchoscopist was required to attempt to intubate all pulmonary segments.

### Measurements and time points

During PDT, visualization of the trachea and quality of ventilation were rated according to a score previously used by our study group (see Table 1) [14]. Each item was rated on a 4-point Likert scale as follows: 1 = very good; 2 = good; 3 = difficult; or 4 = impossible. The quality of ventilation (Table 1, line E) was rated twice: The first rating was obtained before puncture of the trachea (E1), and the second rating reflected the worst ventilation during the PDT (E2). We amended our rating set for the quality of suctioning (F) and handling of the bronchoscope (G). To reduce a potential bias introduced during scoring, all ratings were obtained by an independent physician who observed the PDT but did not participate in the intervention.

**Table 1:** Rating scale for the visualization of tracheal structures and ventilation during percutaneous dilatational tracheostomy

	**Rating**	**1**	**2**	**3**	**4**
A	Identification of thyroid cartilage, cricoid cartilage, first to third tracheal cartilage	Reliable identification	Only cricoid cartilage and tracheal cartilages	Only tracheal cartilage	No vision of tracheal structures
B	Visualization of tracheal circumference	Complete circumference	One-third to two-thirds of circumference	Only small parts of trachea	No vision of tracheal structures
C	Monitoring puncture midline + level below first or second tracheal cartilage	Reliable identification	Midline can be displayed, level uncertain, but below the first tracheal cartilage	Level of puncture uncertain	No vision of tracheal structures
D	Monitoring dilatation anterior wall and pars membranacea (p.m.) visible	Reliable identification	p.m. only	Only small parts of trachea visible, no control of p.m	No vision of tracheal structures
E	Quality of ventilation before puncture and worst ventilation during PDT, respectively	Minute ventilation (MV) as before starting PDT	MV < 2 L/minute or SpO_2_ 80–90% (> 2 min)	MV < 0.5 L/minute or SpO_2_ 70–79% (> 2 min)	MV = 0 or SpO_2_ < 70% (> 2 min)
F	Quality of suction tube for suction of viscous mucus	Easy suction	Suction requires lavage	Suction requires multiple removals of bronchoscope and lavage	Suction not possible
G	Handling of bronchoscope	easy	Impeded, all parts of the bronchial system visible	Intubation of one segment not possible	Intubation of more than one segment not possible

Abbreviations: *MV* Minute ventilation, *PDT* Percutaneous dilatational tracheostomy; *p.m.* Pars membranacea of the trachea, *SpO*_2_ Oxygen saturation as measured by pulse oximetry

Modified from Grensemann et al. [15]. Rating system: 1 = very good; 2 = good; 3 = difficult; 4 = impossible. The quality of ventilation (line E) was rated twice (i.e., before puncture [E1] and to reflect the worst ventilation during tracheostomy [E2])

To assess partial pressure of arterial oxygen (PaO_2_), partial pressure of arterial carbon dioxide (PaCO_2_), and pH values, arterial blood gas (ABG) values were obtained prior to the start of the intervention (time point 1) before skin incision (time point 2) and immediately after insertion of the tracheal cannula (time point 3).

Minute ventilation (MV) before and during tracheostomy, oxygen saturation by pulse oximetry, and capnography (Infinity Delta vital signs monitor; Drägerwerk AG, Lübeck, Germany) were recorded in addition to patients’ demographic parameters and the duration of the intervention at the time points as mentioned above. The Acute Physiology and Chronic Health Evaluation II score [16] and the Sequential Organ Failure Assessment score [17] were recorded on the day of examination as measures of disease severity.

### Outcome parameters

The primary endpoint was the quality of visualization as measured by items A through D on the score, lower values indicating better visualization. Secondary endpoints were the quality of ventilation (scoring items E1 and E2), changes in PaCO_2_, pH, end-tidal carbon dioxide, and PaO_2_, duration of intervention; changes in airway pressure, quality of suctioning and handling of the single-use bronchoscope (rotation of the device, flexion of the tip of the bronchoscope, difficulty to advance and pull back the bronchoscope, items F and G), the combined score (items A through G), and adverse events (bleeding, hypoxemia, injury to trachea and surrounding structures) related to PDT.

#### Costs

For the assessment of the costs of the bronchoscopic procedure, data on costs of bronchoscope procurement, repair, decontamination, and total number of bronchoscopes in use, as well as bronchoscopies per year were obtained from the controlling and accounting department. The procurement costs were split according to the compound value formula with an interest rate of 3% over the useful life-span of the device. Overhead and labor costs (except for decontamination) were deemed similar in both groups and therefore omitted in the analysis.

#### Contamination

Data on the routine contamination analysis of our reusable bronchoscopes were obtained. The reusable bronchoscopes are sampled randomly at least once yearly accordingly to the requirements of the Robert-Koch-Institute (Federal Institute of the German Federal Ministry of Health for Surveillance and Prevention of Diseases), as the responsible governing body. The single-use-bronchoscopes were not sampled for contamination as they were disposed of after their use.

### Sample size

An a priori power analysis for noninferiority testing indicated that a sample size of 46 would be sufficient to detect a difference of 20% in the visualization score (visualization of the trachea as primary end point, noninferiority margin 0.8, lower score values = better visualization) with error probabilities of α = 0.05 and 1 − β = 0.80 (Power Analysis and Sample Size [PASS] version 08.0.6 software; NCSS, Kaysville, UT, USA).

### Randomization

Patients were randomized in a 1:1 ratio using sealed and sequentially numbered opaque envelopes, prepared before start of the study. Patients were randomized and allocated to the respective group immediately before the start of the intervention by an independent physician observing the PDT. No blinding to treatment allocation was deemed feasible.

### Statistics

Microsoft Excel 2016 software (Microsoft Corp., Redmond, WA, USA) was used for data management, and the IBM SPSS Statistics software package (version 25; IBM, Armonk, NY, USA) was used for statistical analysis. The statistical analysis was conducted as published before [15]: We used Welch tests for comparisons of scores. Visualization, ventilation, handling, and combined scores were tested for noninferiority of single use bronchoscopy compared with conventional bronchoscopy. Noninferiority was considered established if the upper limit of the 95% confidence interval (CI) of the difference between the scores of the single use bronchoscopy group of the respective outcome variable did not surpass the mean of the score of the bronchoscopy group by 20% or more (lower scores indicate better performance). The 95% CIs of the mean of the scores were calculated as mean plus and minus the respective value of the t-distribution multiplied by the standard error of the mean calculated as the standard deviation divided by the square root of the sample size. We used linear mixed models with post hoc pairwise comparisons of estimated marginal means for hemodynamic and respiratory variables. In the mixed model analyses, fixed effects of the treatment groups, time points and group × time point, and random intercepts for patients were assumed, employing a variance component covariance matrix. Two-tailed p values < 0.05 were regarded as statistically significant.

## Results

**Fig. 1:**
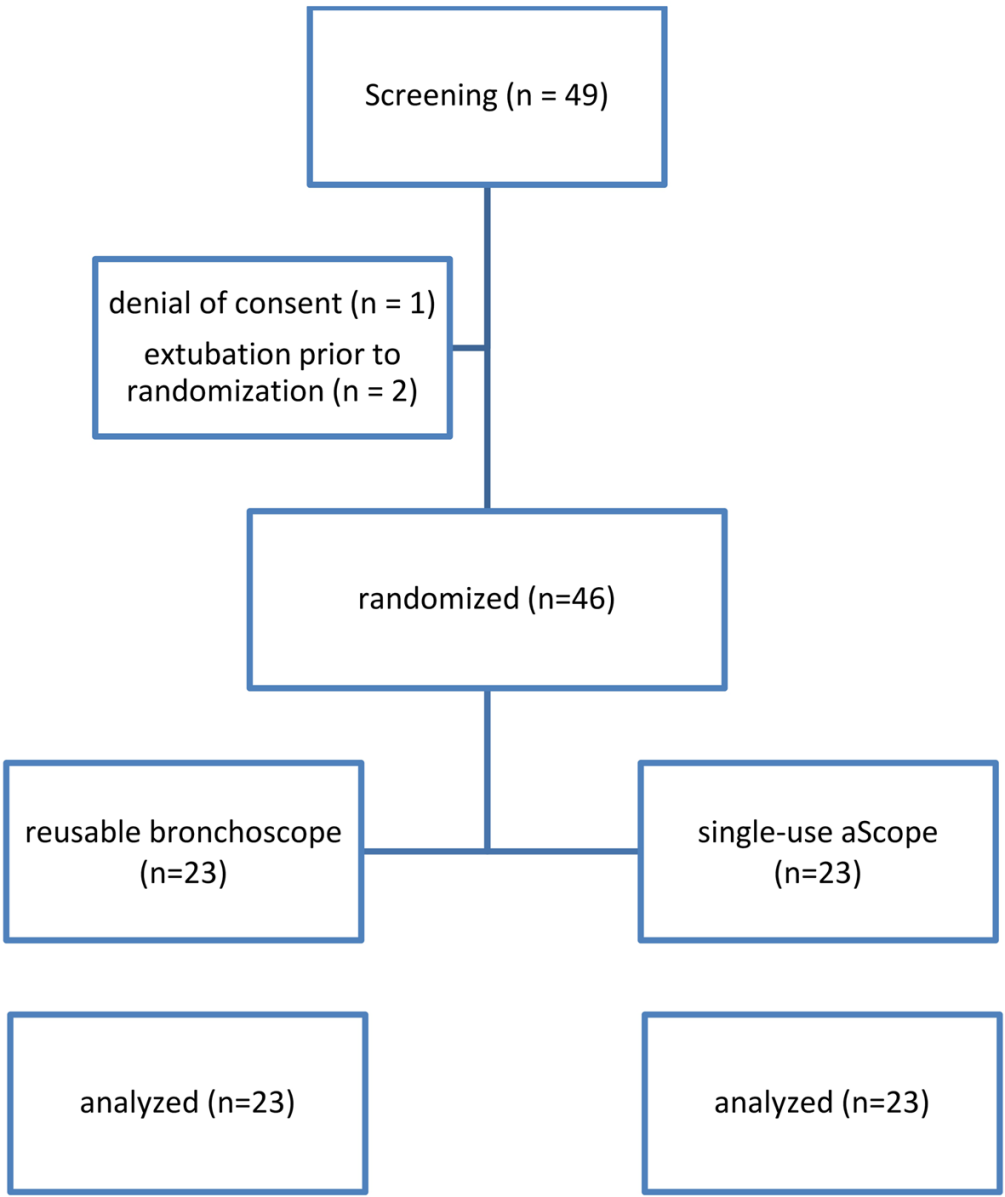
Consolidated Standards of Reporting Trials (CONSORT) diagram

Between May 15 and November 1, 2019, a total of 46 patients receiving PDT for prolonged mechanical ventilation were randomized to either the SUB or the CONV group in a 1:1 ratio (see Fig. 1). Patients’ baseline characteristics are shown in Table 2.

**Table 2:** Patient characteristics

	**SUB (*n* = 23)**	**CONV (*n* = 23)**
Age (years)	63 ± 13	60 ± 13
Height (cm)	174 ± 10	175 ± 10
Weight (kg)	78 ± 17	81 ± 23
SOFA	9 ± 4	9 ± 3
APACHE	22 ± 7	23 ± 7
length of ICU stay (d)	50 ± 52	48 ± 54
**Underlying Condition:**		
general surgery (n)	5	4
vascular surgery (n)	1	1
neurologic (n)	9	10
cardiopulmonary resuscitation (n)	2	3
pneumonia (n)	5	4
acute-on-chronic liver failure (n)	1	1

Abbreviations: *SUB* single-use bronchoscope, *CONV* reusable bronchoscope, *ICU* intensive care unit; data are given as mean ± standard deviation or number, as appropriate

All patients had a Cormack and Lehane score of 1 or 2. The mean procedure duration from skin incision to insertion of the tracheal cannula was 10 ± 6 min (mean ± standard deviation) in both groups (*p* = 0.767). The tubes’ inner diameters were similar in both groups (SUB: 7.7 ± 0.4 mm vs. CONV: 7.8 ± 0.3 mm, *p* = 0.699) and the cross-sectional areas remaining for ventilation during intervention were SUB: 28 ± 5 mm^2^ and CONV: 29 ± 4 mm^2^, *p* = 0.361.

**Fig. 2:**
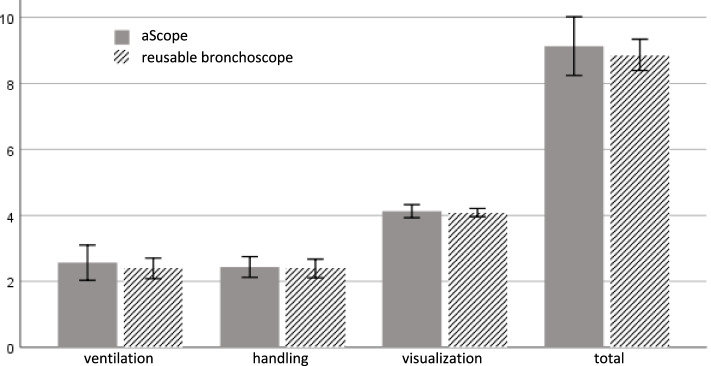
Rating of ventilation, handling, and visualization according to score. Lower scores indicate better performance. Error bars indicate 95% confidence intervals

Noninferiority for visualization (the primary endpoint) was demonstrated for the SUB group. Mean visualization scores were 4.1 (95% CI: 3.9;4.3) for the SUB group vs. 4.1 (4.0;4.2) for the CONV group with a mean difference of 0.0 (-0.2;0.3) (see Fig. 2). Ventilation was rated 2.6 (2.0;3.1) for the SUB group vs. 2.4 (2.1;2.7) for the CONV group, mean difference 0.2 (-0.4;0.8). Handling characteristics were rated at 1.2 (1.0;1.4) for the SUB and 1.3 (1.1;1.6) for the CONV group, mean difference -0.1 (-0.4;0.2). The total scores were 9.1 (8.2;10.0) for SUB and 8.9 (8.4;9.4) for the CONV group, mean difference 0.3 (-0.7;1.2).

No significant difference regarding blood gas analyses and respiratory variables between the groups was found except for an increase in PaCO_2_, etCO_2_, FiO_2_, and PaO_2_, and a decrease in pH over the time course of the intervention (see Table 3). No procedure related adverse events occurred.

**Table 3:** Arterial blood gas analyses and respiratory values

	**Timepoint 1**		**Timepoint 2**			**Timepoint 3**		
	SUB	CONV	SUB	CONV	p	SUB	CONV	p
pH	7.46 [7.43;7.48]	7.43 [7.40;7.46]	7.36 [7.33;7.40] ^a^	7.36 [7.33;7.39] ^a^	0.806	7.29 [7.26;7.31] ^a,b^	7.30 [7.27;7.33] ^a,b^	0.675
paO_2/_ FiO_2_ [kPa]	41 (35;47)	36 (30;42)	56 (50;63)	56 (50;62)	0.828	54 (48;60)	53 (47;59)	0.822
paCO_2_ [kPa]	5.0 [4.4;5.6]	5.4 [4.7;6.0]	6.3 [5.7;6.9]^a^	6.9 [6.2;7.5]^a^	0.216	8.0 [7.4;8.6]^a,b^	8.1 [7.4;8.7]^a,b^	0.925
S_a_O_2_ [%]	95 [92;98]	95 [92;98]	100 [96;103]	100 [96;103]	0.990	96 [93;99]	100 [96–103]	0.104
Peak [hPa]	20 [17;23]	22 [19;25]	22 [19;25]	24 [21;27]	0.223	24 [21;27] ^a^	25 [22;28]	0.572
PEEP [hPa]	7 [6;7]	8 [7;9]	7 [6;8]	8 [7;9]	0.032	7 [6;7]	8 [8;9]	0.019
etCO_2_ [kPa]	4.3 [3.9;4.8]	4.3 [3.9;4.8]	4.7 [4.3;5.1]	4.8 [4.4;5.3]	0.722	5.4 [4.9;5.9] ^a,b^	5.3 [4.8;5.7] ^a^	0.660
V_t_ [ml]	454 [369;539]	516 [432;601]	385 [300;470]	413 [328;497]	0.646	427 [342;512]	412 [327;497]	0.800
MV [l*min^−1^]	10.1 [8.7;11.6]	9.8 [8.4;11.3]	7.4 [5.9;8.8] ^a^	7.6 [6.2;9.0] ^a^	0.805	8.4 [6.9;9.8]	7.6 [6.1;9.0] ^a^	0.406
C [ml*hPa^−1^]	52 [43;60]	45 [36;54]	39 [30;48] ^a^	39 [31;48]	0.971	38 [29;47] ^a^	43 [34;51]	0.430
RR [min^−1^]	22 [19;24]	21 [19;24]	19 [17;21] ^a^	19 [17;21] ^a^	0.912	21 [18;23]	19 [17;21] ^a^	0.306
SpO_2_ [%]	95 [91;99]	94 [91;98]	100 [99;107] ^a^	99 [95;102]	0.086	99 [95;103]	99 [96;103]	0.947

Abbreviations: *SUB* single-use bronchoscope, *CONV* reusable bronchoscope, *paO*_2_ Partial pressure of arterial oxygen, *paCO*_2_ Partial pressure of arterial carbon dioxide, *SaO*_2_ Arterial oxygen saturation, *Peak*: Peak airway pressure, *PEEP* Positive end-expiratory pressure, *etCO*_2_ End-tidal carbon dioxide tension, *V*_*t*_ Tidal volume, *MV* Minute ventilation, *C* Compliance, *RR* Respiratory rate, *FiO*_2_ Fraction of inspired oxygen, *SpO*_2_ Oxygen saturation as measured by pulse oximetry

Data are shown as the mean and 95% confidence intervals, time point 1: before start of intervention, time point 2: before tracheal cannulation, time point 3: after insertion of tracheal cannula. Statistical analysis was done with linear mixed models. p Values in columns indicate differences between single-use and reusable bronchoscope groups at the respective time points

^a^ *p* < 0.05 vs. time point 1

^b ^*p* < 0.05 vs. time point 2

### Cost calculation

In our intensive care units (total 140 beds), 1457 bronchoscopies were performed in 2019 with 24 reusable bronchoscopes equaling 61 bronchoscopies per bronchoscope per year. For the decontamination of one bronchoscope after use, 10 € are billed to our department, including labor costs. From 2017 to 2019, the overall repair costs for 24 bronchoscopes and the corresponding 12 light sources including maintenance and rental bronchoscopes for the temporary replacement of bronchoscopes in repair were 217,959 € equaling 72,653 € per year and thus 50 € per bronchoscopy.

Procurement costs are 14,000 € per bronchoscope and 2,000 € per light source. At a 2:1 bronchoscope to light source ratio, according to the compound value formula at an interest rate of 3% and a useful life span of 10 years per bronchoscope, the proportionate procurement costs are 33 € per bronchoscopy at 61 bronchoscopies per bronchoscope per year.

Therefore, in our institution, the overall costs per bronchoscopy with a reusable bronchoscope are 93 € (repair and maintenance: 50 €, decontamination 10 €, proportionate procurement costs 33 €).

In Germany, the list price of a single-use aScope bronchoscope is 230 € plus the required monitor. At a list price of 2322 € for the monitor, 122 bronchoscopies per year (mimicking the 2:1 bronchoscope to light source ratio) and a useful life span of 10 years, the costs are approximately 2.50 € per bronchoscopy for the monitor according to the compound value formula. Therefore, one bronchoscopy with a single-use bronchoscope adds up to 232.50 €.

### Contamination

None of the reusable bronchoscopes showed any signs of contamination.

## Discussion

The present study aimed at comparing visualization, ventilation, and handling of single-use versus reusable bronchoscopes for PDT. We did not find any significant differences between the two methods concerning visualization, ventilation, and handling, and thus could show non-inferiority for single-use bronchoscopes in the setting of PDT.

Concerning the visualization, the tested single-use bronchoscope uses a camera chip at the tip while the conventional optical fiberscope was connected to a camera head. Although not systematically evaluated in this study, the monitor image of the single-use device with a 480 × 800 pixel resolution appeared sharper than the image of the camera head system. It must be noted that our reusable equipment, being several years in use by now, does not meet the currently available high-definition video standards offered by the industry. However, the visualization during the procedure with either type of bronchoscope was estimated as sufficient by the treating physicians, and we doubt that the use of a current videobronchoscope using a camera chip would have yielded different results. The angle of view of the single-use device was narrower than that of the reusable bronchoscope but this was not noticeable and had no impact on the visualization scores.

Ventilation during the intervention was similar in both groups. After the insertion of the bronchoscopes, PaCO_2_ increased with a consecutive respiratory acidosis with a decrease in pH values as has been shown previously [15, 18, 19]. The main factor leading to hypercarbia was the decrease in the endotracheal tubes’ cross-sectional area. When choosing bronchoscopes for guidance of PDT, the diameter should be considered. It has been recommended to maintain a difference of inner tube diameter and bronchoscope outer diameter of at least 2 mm to maintain a sufficient ventilation and to prevent an auto-positive end-expiratory pressure [20]. Considering the diameter, it may be argued that an even smaller bronchoscope could lead to an improved ventilation with less pronounced hypercarbia, however, the cross sectional area of the suctioning channel decreases as well, possibly preventing the aspiration of mucus or blood from the bronchial tree during the intervention [21]. Therefore, a compromise in necessary to balance ventilation and suctioning. The bronchoscopes used in our study had a similar diameter and no difference existed between remaining cross-sectional areas in the tubes during the intervention. Therefore, a similar performance of the bronchoscopes regarding ventilation was to be expected.

Concerning handling characteristics, both bronchoscopes performed equal. The aScope may be fully inverted to 180° in both up and down direction, while the reusable scope has a travel of 180° only in the up direction and 130° downward. This did not have an influence on our rating scale but the possibility of inverting the bronchoscope in both directions may be useful, i.e. the right upper lobe may also be intubated under sub-optimal conditions as often present in intensive care without the user having to rotate the bronchoscope 180° in his hand. Suctioning was also similar in both groups, as the suctioning channel had a similar diameter in both bronchoscope models.

There was no difference between both groups concerning changes in blood gas analysis during the procedure either. But this was not to be expected as both bronchoscopes used had nearly the same diameter and there was no significant difference between the execution times of the procedure.

Our data revealed higher costs per procedure when single-use bronchoscopes were used in our institution. The costs of bronchoscopy depend on many factors as frequency of uses per device, useful device lifespan, maintenance costs, and decontamination costs. *Châteauvieux *et al*.* recently reported on similar costs as in our institution [22]. The authors calculated a certain number of bronchoscopies for their institution under which the use of SUB was less expensive; however, above that, reusable bronchoscopes were more economical. For reusable bronchoscopes, the costs per bronchoscopy decreased with a higher number of interventions. In contrast, a recent meta-analysis on cost effectiveness of single-use versus reusable bronchoscopes for PDT claimed single-use devices more cost effective [23], but disinfection costs in this analysis were far higher than in our institution and the main savings were achieved when additional expenses for the treatment of pneumonia due to the risk of cross-contamination, i.e. insufficient disinfection and spread of pathogens from one to the next patient, were included.

In our analysis, we did not include an estimate for additional treatment expenses due to possible cross contamination induced infections. The incidence of cross contamination is difficult to predict, and data published on this topic have often revealed systematic problems in the decontamination/disinfection process, non-adherence to reprocessing guidelines, or mechanical damage to the bronchoscopes [24, 25]. Therefore, contamination rates might easily be overestimated, and routine screening of our bronchoscopes showed no indication of contamination. Adherence to reprocessing guidelines i.e. including pre-cleaning with enzymatic detergent and mechanical testing seems of utmost importance to prevent cross contamination [26]. Of note, transmission of pathogens during bronchoscopy may be unrelated to the bronchoscope itself and e.g. contaminated lubricant has been reported [27].

Nevertheless, the possibility of cross contamination should not be neglected and has been estimated at around 3% with a 21% risk of subsequent infection by some authors [2, 3]. Follow up costs for the treatment of healthcare associated pneumonia have been estimated at around 3000€ with an excess mortality of 6% [28, 29]. Considering these data, single use bronchoscopes could provide economic benefits. We suggest that each institution revisits their bronchoscope reprocessing, monitors closely for any signs of contamination, and calculates their risks for induced infections. With this information, a data-based decision can be made upon cost-effectiveness of single-use bronchoscopes.

PDT was chosen for our study because this procedure is well standardized and thus allows a good comparability between the two types of bronchoscopes. The feasibility of PDT with guidance from a single-use bronchoscope has been shown previously, but only in a case series with bronchoscopes without a suctioning channel that are obsolete by now [30]. Although bronchoscopy permits for visualizing the site of puncture and correct positioning of the dilation devices, it must be noted that routine bronchoscopy remains controversial as some authors point out the risks of hypercarbia with consecutive respiratory acidosis and claim individual risk assessment for patients [15, 31]. In comparison to PDT, data concerning ventilation and risk of hypercarbia show favorable results for surgical tracheostomy [19] and presumably other methods not reducing the endotracheal tubes’ cross section during the intervention, i.e. guidance by sonography [32] or an endotracheal tube mounted camera [14]. Nevertheless, bronchoscopy is used in approximately 70% of all PDT procedures, especially in Europe [7].

Considering PDT, periprocedural damage to the bronchoscope has been reported if the bronchoscope is hit by the puncture needle during cannulation of the trachea [33, 34]. In our institution, no defect in any bronchoscope was caused by PDT during the assessed three years, presumably because only experienced attending intensivists performed tracheostomies in our institution. This may explain the low costs for bronchoscope repairs in our cohort.

Our study has the following limitations: Our primary outcome measure was based on Likert scales. Although the ratings were based on objective parameters and obtained by an independent physician, the examiners’ expectations and opinions may have influenced the scoring, thus introducing a bias. Furthermore, no blinding of the examiner to the type of intervention was done. The cost analysis is error-prone because several assumptions especially concerning the risk of cross-contamination must be made.

## Conclusion

In our study, the performance of single-use bronchoscopes for PDT concerning visualization of relevant anatomic trachea structures, ventilation, handling, and performance of the suction tube was non-inferior to reusable bronchoscopes. Single-use bronchoscopes may be advantageous if high rates of cross-contamination are present or only few procedures per device are performed, thus increasing the per procedure cost of reusable bronchoscopes. Further studies are needed to quantify the rates of infection transmission due to cross contamination. We suggest that each institution revisits their own data on infection rates and performs a cost analysis of the procedure costs of bronchoscopy.

## Data Availability

The datasets used and/or analyzed during the current study are available from the corresponding author on reasonable request.

## Abbreviations

CONV: Conventional, reusable bronchoscope
PDT: Percutaneous dilatational tracheostomy
SUB: Single use bronchoscope
